# Successful Management of Kratom Use Disorder With Buprenorphine and Naloxone

**DOI:** 10.7759/cureus.41146

**Published:** 2023-06-29

**Authors:** Martin Arhin, Julian Mobley, Hamad Hamad, Paul Remick

**Affiliations:** 1 School of Medicine, The University of North Carolina at Chapel Hill, Chapel Hill, USA; 2 Family Medicine, Geisinger Commonwealth School of Medicine, Scranton, USA

**Keywords:** opioid use, methadone clinic, buprenorphine and naloxone, opioid taper, kratom addiction

## Abstract

Kratom is an unregulated herbal supplement that is growing in popularity in the United States. Its primary active ingredients, mitragynine, and 7-hydroxymitragynine, are partial agonists that act on mu- and delta-opioid receptors in the central nervous system, producing analgesia and a sense of euphoria. Kratom use can lead to addiction and adverse side effects, such as seizures, hallucinations, and coma. This case report presents a male in his 40s with a history of kratom use, who presented to the clinic seeking help for his addiction. The patient had been using kratom for several years to self-medicate for his anxiety and depression and gradually developed a kratom addiction. The patient was successfully treated with buprenorphine/naloxone, which helped alleviate his withdrawal symptoms and allowed him to abstain from kratom. This case underscores the growing issue of kratom addiction in the United States and the critical need for physician awareness in treating withdrawal.

## Introduction

Kratom, also known as *Mitragyna speciosa*, is a member of the coffee family and has been used as a form of traditional medicine in Southeast Asia, particularly in Thailand, Malaysia, and Indonesia, for centuries [1]. Traditionally, it was consumed by chewing or brewing the leaves into tea [1]. Its use as a recreational drug can be traced back to the early 19th century when it was first used to relieve fatigue, pain, and enhance productivity [2]. It is also used as a social drug, consumed during ceremonies and social gatherings [2]. Kratom's use continued to spread throughout Southeast Asia, and by the late 20th century, it had also gained popularity in Western countries.

Kratom's primary active ingredients, mitragynine (MIT) and 7-hydroxymitragynine (7-HMG), act on the same receptors in the brain as opioids, producing analgesia and a sense of euphoria [3]. The mu-opioid receptor is the primary site of action for kratom's analgesic and rewarding effects. MIT and 7-HMG bind to this receptor, producing a range of effects like those of traditional opioids [3]. However, unlike traditional opioids, kratom's alkaloids act as partial agonists at the mu-opioid receptor, which means that they activate the receptor to a lesser extent than full agonists, such as morphine or fentanyl. This partial activation results in a less-intense opioid-like effect than full agonists, which can reduce the risk of respiratory depression, a potentially life-threatening side effect of opioid use [4]. 

Kratom's alkaloids also interact with other receptors in the brain, including the kappa-opioid and alpha-2 adrenergic receptors. These interactions can further enhance its pain-relieving properties and may contribute to the drug's sedative effects [5]. The kappa-opioid receptor is involved in the regulation of pain and stress responses, and the alpha-2 adrenergic receptor plays a role in the regulation of blood pressure and heart rate. The interactions of kratom's alkaloids with these two additional receptors may contribute to the drug's overall pharmacological effects, such as tachycardia, cardiac arrest, and analgesia [6,7]. Drug effects vary depending on the dose, with low doses producing a stimulant-like effect and higher doses producing sedative effects. Due to its opioid-like effects, kratom is used to manage chronic pain, anxiety, and depression with high rates of user self-reported effectiveness [1]. Despite its potential benefits, kratom is also known to have addictive properties, and its use has been associated with adverse effects, such as confusion, hallucinations, and seizures [2].

The legality of kratom use varies widely across the globe. In many Southeast Asian countries where kratom is indigenous, such as Thailand and Malaysia, it is illegal to possess or use the plant [8]. However, in other countries such as Indonesia, it is legal to cultivate and sell kratom. In Europe, kratom's legality is similarly varied. In some countries, such as Sweden and Finland, it is classified as a medicinal product and can only be obtained with a prescription. In other countries, such as Germany, kratom is legal to purchase and use.

In recent years, the use of kratom has grown rapidly in the United States. This surge has been accompanied by an increase in scientific research into the plant's effects and potential health risks, such as the increasing number of case reports [8]. The opioid epidemic in the United States has also had a significant impact on the growing demand for kratom. With the tightening of opioid medication prescribing guidelines, patients with opioid dependence are increasingly seeking alternatives to traditional opioids, such as kratom, to manage their addiction [9]. Additionally, some individuals who suffer from chronic pain or opioid withdrawal have turned to kratom as a potential alternative or supplement to traditional opioid medications. Kratom's expanding use in the Western markets is a result of its legal status and easy accessibility [9].

The Drug Enforcement Administration (DEA) previously announced its intent to classify kratom as a Schedule I controlled substance, which would have made it illegal to possess, use, or distribute. However, following a public outcry and a congressional letter opposing the proposed scheduling, the DEA ultimately withdrew its intent to schedule kratom. Currently, kratom is legal in most states, although some have banned its use or have pending legislation to do so [10]. The US Food and Drug Administration (FDA) has not approved kratom for any medical use and has issued warnings about the potential dangers of kratom use.

Despite this, kratom is often considered a safer alternative to opioids, but the efficacy of the substance for such purposes remains highly questionable. More research is needed to establish a conclusive solution [1]. The relationship between the opioid epidemic and kratom use remains a topic of ongoing research and debate in the medical and scientific communities [9]. This case report underscores the need for continued research into the potential risks and benefits of kratom use. 

## Case presentation

A male in his mid-40s presented to his primary care physician seeking help for his kratom addiction. He has a history of anxiety disorder, major depressive disorder, asthma, obesity, and chronic obstructive pulmonary disease. While he had not experienced any recent episodes of depression, his anxiety was not well controlled. Additionally, he had a past history of substance misuse of cocaine and alcohol that did not meet the criteria for substance use disorder and opioid use disorder. He reported occasionally using marijuana and was prescribed varenicline for his nicotine dependence. His current medications included bupropion, sertraline, lorazepam as needed, and ipratropium bromide/albuterol sulfate. 

The patient was introduced to kratom 12 years ago by his brother for relaxation, stress relief, and as a substitute for oxycodone. He gradually increased his dose up to 40g of powder per day from an initial 6g/daily. He purchased kratom online and at a local gas station, and his weekly expenditure on the substance ranged from $100 to $600. Over time, he spent less time with family and friends, engaged in fewer recreational activities, and spent more time using kratom to relax at home. He realized that he was dependent on the substance when he experienced withdrawal symptoms such as diarrhea and anxiety when he skipped or reduced his use of the substance. He attempted to self-treat his addiction by decreasing his dose but experienced severe withdrawal symptoms, including stress, headaches, diarrhea, nausea, and dizziness. His inability to quit kratom prompted him to seek medical help from his primary care physician. This was his first time seeking help from a physician. The patient was provided with the opportunity to connect with substance abuse clinics and other self-help groups, but he preferred pharmacological treatment. 

The patient's treatment plan was as follows:

• Week 1: The patient was started on buprenorphine hydrochloride (HCl)-naloxone HCl at 2-0.5mg, at two pills every four hours. He noted improvement of symptoms and no withdrawal symptoms except for a "runny nose." No other upper respiratory symptoms were present.

• Weeks 2-4: His dose was increased to 8-2mg. He took 1-1/4 tablets for three days, one tablet for three days, and 3/4 tablets for three days. He initially felt mild anxiety, but symptoms improved over the trial period and his rhinorrhea resolved. The dose was subsequently decreased to 2-0.5mg, and he was instructed to take three tablets daily for two days, two tablets daily for three days, 1-1/2 tablets daily for three days, and one tablet per day thereafter. A follow-up was scheduled for two weeks.

• Weeks 5-6: The patient experienced withdrawal symptoms when attempting to reduce the dose further. He continued his prior regimen of one tablet daily for another two weeks.

• Weeks 7-11: The patient felt ready to take another reduction of his dose. The dose was reduced to a 2mg tablet that was alternated with a 1mg tablet daily for two weeks. This dose was continued for three weeks. 

• Weeks 12-15: The dosage was reduced to one tablet, alternating with 1/2 tablet daily for two weeks and then 1/2 daily after the two-week trial for a total of one month.

• Weeks 16-19: The dose was changed to 2-0.5mg 1/2 strips daily for four days, then alternating with 1/4 strip daily for seven days, and then 1/4 strip daily for a total duration of a month. During this time, he reported mild fatigue but did not attribute it to his dose change.

• Weeks 20-21: The dose was reduced to 2-0.5mg 1/8 strips daily for two weeks. He was still experiencing some fatigue.

• Weeks 22-23: The patient was tapered off buprenorphine HCl-naloxone HCl. 

During the three-week follow-up appointment, the patient still reported mild fatigue which may be due to kratom withdrawal. Currently, the patient is no longer taking kratom.

## Discussion

Kratom addiction is a growing concern due to the substance's increasing popularity and potential for abuse. The estimated epidemiology is 0.8-1.5% of the United States' population, which corresponds to about 3-5 million users [11]. A lack of regulation has contributed to its growth. Consequently, the market is filled with adulterated products, such as krypton, which contains O-desmethyltramadol and has been associated with nine deaths [12]. The FDA identified 44 deaths associated with kratom use in 2017. Likewise, the Center for Disease and Control (CDC) identified 25 deaths related to kratom use between 2016 and 2017. The deaths were believed to be due to adulterated kratom products and use of other substances [13].

A kratom dose of 5g of powder or more is a high dose with the risk of toxicity becoming more likely in doses exceeding 8g [14]. The patient had developed tolerance after years of use and up to 40g doses per day. He experienced opioid-like withdrawal symptoms, such as diarrhea, upon decreasing the dose and denied any symptoms of opioid toxicity. The most common adverse side effects associated with kratom use were agitation (18%), tachycardia (16.9%), drowsiness (13.6%), vomiting (11.2%), and confusion (8.1%) [2]. Serious side effects were seizures, hallucinations, respiratory depression and arrest, and coma [2].

Currently, there is no established evidence-based consensus for the treatment of kratom addiction and withdrawal, leaving individual providers to decide on the appropriate course of action [15]. However, some approaches have shown effectiveness in treating kratom addiction. Some approaches that are commonly used to treat opioid withdrawal have also been used successfully to treat kratom withdrawal, including buprenorphine and clonidine [15]. Our patient was successfully treated for his kratom addiction over the course of six months with buprenorphine and naloxone. Our approach to treating kratom addiction was to manage it medically, such as opioid addiction. We focused on prescribing dosing that would prevent withdrawal symptoms, so additional medications would not need to be prescribed to manage withdrawal symptoms. The patient experienced initial rhinorrhea and mild fatigue during the six-month treatment period. He did not experience other common withdrawal symptoms, such as muscle aches, sweating, abdominal cramping, diarrhea, nausea, vomiting, mydriasis, and seizures. 

## Conclusions

The case presented demonstrates the growing issue of kratom addiction in the United States and the need for physician awareness and effective treatment strategies. Kratom, an unregulated herbal supplement, gained popularity due to its opioid-like effects. However, its use can lead to addiction and adverse side effects, including seizures, hallucinations, and coma. As the prevalence of kratom addiction increases, healthcare professionals must recognize the signs and symptoms of this substance misuse. The patient in this case had a history of self-medication for anxiety and depression, which eventually led to a kratom addiction. We recognized the patient's dependency on kratom and subsequently implemented a treatment plan utilizing buprenorphine/naloxone, which effectively alleviated withdrawal symptoms and supported the patient's abstinence from kratom. We suggest that this drug combination may be a potential treatment for kratom addiction. Further research is needed to understand the mechanisms underlying kratom addiction and develop more targeted and effective treatments. 
